# Role of Extracellular Hemoglobin in Thrombosis and Vascular Occlusion in Patients with Sickle Cell Anemia

**DOI:** 10.1155/2011/918916

**Published:** 2010-12-27

**Authors:** Zhou Zhou, Molly Behymer, Prasenjit Guchhait

**Affiliations:** Thrombosis Research Division, Cardiovascular Research Section, Department of Medicine, Baylor College of Medicine, One Baylor Plaza, N1319, Houston, TX 77030, USA

## Abstract

Sickle cell anemia (SCA) is a common hemolytic disorder caused by a gene mutation in the *β*-globin subunit of hemoglobin (Hb) and affects millions of people. The intravascular hemolysis releases excessive amount of extracellular hemoglobin (ECHb) into plasma that causes many cellular dysfunctions in patients with SCA. ECHb scavenges NO which promotes crisis events such as vasoconstriction, thrombosis and hypercoagulation. ECHb and its degradation product, heme, are known to cause oxidative damage to the vessel wall and stimulate the expression of adhesive protein ligands on vascular endothelium. Our study shows that ECHb binds potently to VWF—largest multimeric glycoprotein in circulation—through the A2-domain, and significantly inhibits its cleavage by the metalloprotease ADAMTS13. Furthermore, a subpopulation of VWF multimers bound to ECHb exists in significant amount, accounting for about 14% of total plasma VWF, in SCD patients. The Hb-bound VWF multimers are resistant to ADAMTS13, and are hyperactive in aggregating platelets. Thus, the data suggest that Hb-bound VWF multimers are ultralarge and hyperactive because they are resistant to the protease. The Hb-bound VWF multimers are elevated parallely with the level of ECHb in patients' plasma, and is associated with the pathogenesis of thrombosis and vascular occlusion in SCA.

## 1. Introduction

Sickle cell anemia (SCA) is a hemolytic disorder first described by Herrick in 1910 [1]. In 1949, Pauling and his team first demonstrated the molecular basis of SCA, showing that the disease is caused by a small difference in the molecular structure of hemoglobin, an oxygen-carrying protein in plasma [2]. In 1957, Ingram discovered that the disease was caused by a single amino acid substitution (Glutamic acid → Valine) in the *β*-globin subunit of hemoglobin [3]. 

Sickle cell anemia affects millions of people worldwide and is associated with significant morbidity and mortality. An estimated 2% of the world's population carries genes responsible for SCA. Each year about 300,000 infants are born with SCA, including more than 200,000 cases in Africa [4]. More than 33% of deaths in SCA patients are caused by vascular occlusions and related crises, such as strokes and transient ischemic attacks [5, 6]. It is estimated that stroke alone results in 20% mortality in such patients between the ages of 5 to 10 years, with 70% of those patients having a motor deficit and significant neurocognitive deficits; 70% have a recurrent stroke within the next 3 years [7, 8].

It is known that under hypoxic conditions, deoxygenation triggers a hydrophobic interaction between the mutated hemoglobin (HbS) molecules, resulting in the polymerization of HbS and sickling of the RBCs. Sickling alters the cell membrane properties, which reduce cellular flexibility and lead to unusual cell adherence to vascular endothelium [5, 6]. Studies further suggest that sickling alters the RBCs' membrane properties including a significant expression/exposure of different adhesive molecules, which mediate the adhesion of sickle RBCs to endothelium and subendothelial matrix [9–16]. Previous studies, including our own, suggest that exposure of a membrane lipid such as sulfated glycosphingolipid or sulfatide on sickle RBCs promotes sickle cell adhesion to endothelial ligands (such as *α*_v_ *β*_3_ and ULVWF) and subendothelial matrix proteins (such as VWF, TSP, LN and FN) [17–19]. Furthermore, the data also demonstrate that sickle RBCs express a significant quantity of phosphatidylserine (PS) on the cell surface and promote the adhesion to the endothelium through binding to *α*_v_ *β*_3_ [20]. Besides the increase of expression/exposure of adhesive molecules on sickle RBCs, which is associated with increased cell adhesion to the vessel wall, sickling also causes the release of excessive extracellular hemoglobin (ECHb) into plasma from the sickle RBCs during intravascular hemolysis.

## 2. Extracellular Hemoglobin Causes Cellular Dysfunctions

Hemoglobin is a heme-containing globular protein in RBCs that can deliver oxygen and remove carbon dioxide to/from cells and tissues. In hemolytic conditions such as SCA, RBCs release an excessive amount of ECHb into plasma, ranging from 20–330 *μ*g/mL in patients, which can exceed 410 *μ*g/mL during vasoocclusive crisis [21–23]. Upon release, ECHb forms a complex with the hemoglobin-scavenger, haptoglobin, in circulation and is cleared by binding to CD163 on macrophage or leukocyte surfaces [24, 25]. Haptoglobin can bind approximately 70–150 *μ*g/mL of ECHb depending on the haptoglobin allotype [26]. Once the capacity of haptoglobin is exceeded, an elevated level of ECHb accumulates in plasma, causing many cellular dysfunctions. In severe hemolytic diseases such as PNH and SCA, serum haptoglobin is typically undetectable [27].

The accumulation of excessive ECHb in plasma intensifies the consumption of endogenous nitric oxide (NO), resulting in several cellular dysfunctions such as vasoconstriction and systemic and pulmonary hypertension in patients with hemolytic disorders including SCA. It has been shown that the ECHb could scavenge NO, an important endogenous vasodilator, and impair the vasodilatory response to infusions of the direct-acting NO donor to patients [28]. Consistently, patients with plasma ECHb levels higher than 100 *μ*g/ml have shown an 80% reduction in NO-dependent blood flow responses [28]. Other *in vivo* studies further show that nitroglycerin-induced vasodilation is impaired in SCA patients [29] and the diminished vasomotor response to NO donors is observed in transgenic SCA mice [30]. 

Excessive ECHb also causes other cellular dysfunctions in SCA. The elevated ECHb in patients' plasma impairs renal function. Plasma ECHb is normally filtered through the glomerulus and actively reabsorbed in proximal tubule cells where it is catabolized with release of iron in the form of hemosiderin. When the capacity of kidney's reabsorption is exceeded, crises events such as renal dysfunction and failure occur [31]. Second, excessive plasma ECHb may also contribute to platelet activation and thrombosis. An *in vitro* experiment shows that the addition of ECHb to human serum at concentrations of 0.2–2 mg/mL dose-dependently inhibits the activity of metalloprotease ADAMTS13, an enzyme critical in limiting platelet thrombus formation [32]. It is also suggested that the major untoward effects of ECHb on platelet function are most likely mediated by NO scavenging. NO has been shown to inhibit platelet aggregation, induce disaggregation of aggregated platelets, and inhibit platelet adhesion through increasing cyclic guanine monophosphate (cGMP) levels [33]. In addition, NO is also known to interact with components of the coagulation cascade (such as factor XIII and fibrin) to downregulate clot formation [34, 35]. Thus, NO scavenging by ECHb or the reduction of NO generation may result in an increase in intravascular coagulopathy.

## 3. Extracellular Hemoglobin Blocks Cleavage of VWF Multimers

Our investigation shows that excessive ECHb significantly inhibits the cleavage of VWF [23], a multimeric protein in circulation that normally serves in hemostatic functions. However, under pathophysiological conditions such as in SCA patients, the hyperactive VWF multimers play a significant role in cell adhesion and prothrombotic complications. This is particularly significant given that VWF multimers secreted from endothelial cells or platelets may accumulate as ultralarge (UL) multimers in plasma if not properly cleaved by the metalloprotease ADAMTS13. As evident in SCA, inflamed endothelium constitutively secretes ULVWF, maintaining a high VWF antigen level in plasma. Studies, including our own, suggest that elevated VWF levels, particularly ultralarge multimers, exist in SCA patients' plasma, and are associated with increased sickle cell and platelet adhesion to vascular endothelium [23, 36–39]. Several studies have also suggested that ULVWF multimers contain all the determinants necessary for blood cells (including platelets, sickled-RBCs, and neutrophils) to tether and stably adhere to endothelium spontaneously [40–42].

**Figure 1:**
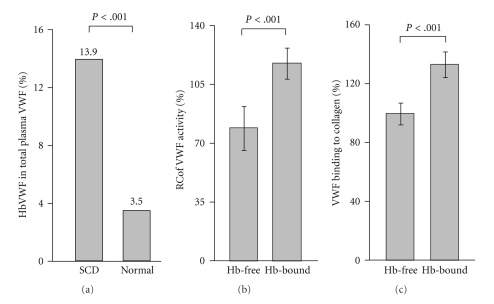
Hb-bound VWF multimers are prevalent in SCD patients and are hyperactive. (a): the VWF was purified from plasma (1 mL) of SCD patients or normal individuals by a Superose gel filtration column. The 1st protein peak (at 280 nm UV) was collected for VWF. Further, the Hb-bound VWF was isolated from above VWF fraction using an Ni-NTA affinity column. The VWF antigen level was measured using a commercial kit. The Hb VWF multimers existed as about 14% of total VWF in patients compared to only about 3.5% in normals (*n* = 5). (b): the ristocetin cofactor VWF activity assay shows that the Hb-bound VWF multimers are 35% more activity than the Hb-free counterparts. (c), The collagen binding assay also shows that the Hb-bound VWF multimers are 33% more adhesive to collagen than the Hb-free multimers.

Considering the implicated role of VWF in SCD pathophysiology, we have investigated the function of the plasma metalloprotease ADAMTS13 that determines the length and activity of VWF. Though SCD patients have an elevated level of higher molecular weight or ultralarge VWF multimers in plasma than normal, they have a very mild [23] or no [36] deficiency in ADAMTS13 activity. This is important given that ULVWF multimers freshly secreted from endothelial cells are accumulated in plasma due to impaired cleavage by ADAMTS13, as seen in patients with thrombotic thrombocytopenic purpura (TTP). Severe TTP patients have very low ADAMTS13 activity (<5% of normal) caused by a genetic mean or autoantibody inhibition, resulting in the accumulation of ULVWF multimers and the development of thrombotic microangiopathy [43–45]. We therefore suggested that such a mild deficiency in ADAMTS13 in SCD patients (70% of normal) is probably insufficient to cause any similar effects on the VWF axis. Consistent with that notion, we have shown that ECHb does interact with VWF to inhibit its cleavage by ADAMTS13, and the mechanism is independent of the metalloprotease activity [23]. We have further demonstrated that ECHb binds to the ADAMTS13 cleavage site on the A2 domain of VWF multimers to block its cleavage by the metalloprotease. The study shows that the presence of 100 *μ*g/mL of ECHb in buffer completely inhibited the VWF cleavage by ADAMTS13 under physiological flow shear conditions [23]. Since the SCA patients have elevated ECHb in plasma, as estimated up to 410 *μ*g/mL during their vasoocclusive crisis [21–23], we speculated that excessive ECHb could impair VWF cleavage by ADAMTS13 *in vivo* and promote accumulation of hyperactive VWF multimers in patients' plasma. Accordingly, we have isolated a subpopulation of VWF multimers from plasma that are bound to ECHb [46]. The Hb-bound VWF multimers exist in SCA patients' plasma, accounting for 14% of total plasma VWF, which is about 4-folds more than normal individuals (Figure 1). Though the interaction between ECHb and VWF and their relationship *in vivo* is still not clearly understood, our data have shown that increased Hb-bound VWF multimers parallely coexisted with the high ECHb level in patients' plasma (mean ± SE ~ 263.8 ± 41.7 (SCA) versus 46.2 ± 9.6 (Normal, *n* = 5)) [46]. Furthermore, we have observed that ECHb preferentially binds to the endothelial cell-purified ULVWF over the plasma-purified VWF multimers. It is possible that ECHb binds to ULVWF multimers that are freshly released into plasma and have their A2-domains exposed. As evident, the A2-domain of ULVWF multimers has unique features such as a lack of protection by disulfide bonds within VWF and low resistance to unfolding that help the domain to be exposed easily [47]. When exposed to intravascular hydrodynamic shear forces, the tensile force on the ULVWF multimers increases by the square of the multimer length, providing an efficient mechanism for unfolding of the A2 domain on ULVWF [48]. The similar mechanism in which shear force induced exposure of the A2 domain in freshly released (UL)VWF multimers could probably facilitate the binding of ECHb to VWF, which prevents the cleavage of ULVWF multimers by ADAMTS13. As expected, we found that Hb-bound VWF multimers are 30–35% more adhesive to platelets and subendothelial matrix collagen and less cleavable by ADAMTS13 compared to their Hb-free counterparts [46].

**Figure 2:**
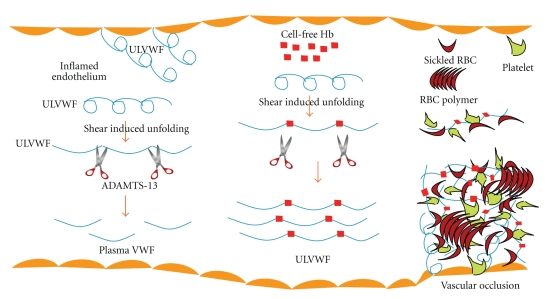
The schematic diagram shows that upon activation of the vascular endothelium, ultralarge VWF is released and is cleaved by ADAMTS13 to smaller fragments that circulate in plasma as an inactive form. Under pathophysiological conditions such as SCA, excessive extracellular Hb blocks the cleavage of a subpopulation of VWF multimers. The uncleaved Hb-bound VWF multimers accumulate in plasma, which are hyperactive in binding to platelets or sickled/fragmented-RBCs to promote cell adhesion and events such as thrombosis and vascular occlusion.

We have speculated that elevated plasma Hb-bound VWF, parallely with increased ECHb, might play an important role in the culmination of blood cell (including platelets, sickle RBCs, and neutrophils) adhesion to vascular endothelium and development of related crises events such as thrombosis, thromboembolism, ischemic strokes, and myocardial infarctions in patients with SCA. This mechanism is not only limited to SCA, but also occurs in other hemolytic disorders including thalassemia, and hemolytic uremic syndrome. This mechanism is shown through a schematic diagram in Figure 2.
